# Carbon Nanotubes: Probabilistic Approach for Occupational Risk Assessment

**DOI:** 10.3390/nano11020409

**Published:** 2021-02-05

**Authors:** Andrea Spinazzè, Carolina Zellino, Francesca Borghi, Davide Campagnolo, Sabrina Rovelli, Marta Keller, Giacomo Fanti, Andrea Cattaneo, Domenico M. Cavallo

**Affiliations:** Dipartimento di Scienza e Alta Tecnologia, Università degli Studi dell’Insubria, Via Valleggio, 11–22100 Como, Italy; carolinazellino@gmail.com (C.Z.); f.borghi2@uninsubria.it (F.B.); davide.campagnolo@uninsubria.it (D.C.); sabrina.rovelli@uninsubria.it (S.R.); mkeller@uninsubria.it (M.K.); giacomo.fanti@uninsubria.it (G.F.); andrea.cattaneo@uninsubria.it (A.C.); domenico.cavallo@uninsubria.it (D.M.C.)

**Keywords:** SWCNTs, MWCNTs, nanotechnology, occupational exposure, occupational health, engineered nanomaterials

## Abstract

In this study, the occupational risk assessment of carbon nanotubes (CNTs) was performed by means of a probabilistic approach. Chronic and subchronic inhalation exposure studies were retrieved during the hazard identification phase of the study. These studies were then used to obtain a guidance value (BMC_h_, expressed as a lognormal distribution with geometric mean ± geometric standard deviation = 10.0 ± 4.2 µg/m^3^) for occupational inhalation exposure to CNTs. An exposure scenario was selected from the scientific literature: three different work events (WEs) related to the production of conductive films were considered: (WE1) manufacturing of single walled carbon nanotubes films during normal operation using local exhaust ventilation (LEV); (WE2) manufacturing of SWCNT film without LEV; and (WE3) cleaning of one of the reactors. For each WE, a probability distribution function was applied, considering exposure expressed as mass concentration, as derived from three different measurement techniques. The ratio of the exposure and the BMC_h_ distributions (i.e., the risk characterization ratio—RCR) was used to calculate the probability of occurrence of a relevant occupational risk. All the considered WEs indicated the presence of a risk (i.e., RCR distributions ≥ 1); however, only WE2 resulted in a statistically significant level of risk.

## 1. Introduction

Reliable procedures for risk assessment (RA) need to be constantly implemented to evaluate the possible health impacts of engineered nanomaterials (NMs). Although in theory the RA methodology is well established for other types of chemicals, the RA process for the NMs is challenging on a practical level [1,2]. Among the major limitations concerns the availability of reliable information on NM to properly set the hazard assessment (i.e., hazard identification and dose–response relationship evaluation) and the exposure assessment phases of the RA [1,3]. Applying a probabilistic approach (in which all the parameters can be defined as value distributions) instead of the commonly adopted deterministic approach (which uses single values for parameters) allows us to consider and determine uncertainties (e.g., those related to toxicological data and measured exposure) and to define clearly the likelihood of risk [4,5]

The aim of the present study is to apply a probabilistic approach in a case study, to perform a risk assessment for an NM in selected occupational settings. The classical four steps in the RA process (1.) hazard identification, (2.) dose–response assessment, (3.) exposure assessment and (4.) risk characterization) were followed [6]. The same approach for occupational RA of NMs was firstly suggested by Tsang et al. [4] for titanium dioxide nanoparticles and later applied by Spinazzè et al. [5] for graphene nanoplatelets. In this study, the probabilistic RA approach was applied to carbon nanotubes (CNTs); CNTs are graphene sheets (comprising sp^2^-hybridized carbon bonds) structured in cylindrical tubule with diameters from <1 nm to 10 s of nm. Nanotubes could be multiwalled (MWCNTs: multiwalled carbon nanotubes) or single-walled (SWCNTs: single-walled carbon nanotubes) structures. CNTs possess characteristics (e.g., excellent mechanical properties, high chemical stability, very high electrical conductivity and large specific surface ratio), making them of interest in nanoscience and in several emerging nanotechnology applications (e.g., semiconductors, flame-retardants and fuel systems in automotive and aerospace industries) [7,8].

Available studies outlined that (i) some adverse effects (similar to the toxicological endpoints identified by means of in vivo animal studies) have been identified for humans [8]; (ii) fibrosis and pulmonary inflammation could be potential outcomes in exposed populations (depending on exposure level and duration); (iii) fibrosis, inflammatory and oxidative stress biomarkers have been shown to be strongly associated with CNT exposure. However, to date: (i) no noticeable adverse effects in workers exposed to CNT have been reported; (ii) the lack of environmental or internal dose‒exposure measurements prevents the identification of suitable dose‒response relationships; and overall (iii) conclusive information on the hazards posed by CNTs to human health is still limited to reach definitive conclusions about the potential hazards in a risk assessment procedure [3].

## 2. Materials and Methods

### 2.1. Hazard Identification

Recent peer-reviewed studies concerning in vivo toxicology tests for both SWCNTs and MWCNTs were selected. Only chronic and subchronic inhalation exposure studies were considered, as these conditions were assumed as the most relevant potential exposure in the occupational settings. Different dose-metrics (mass concentration and fiber counts) were considered.

### 2.2. Dose–Response Assessment

To estimate a health-based guidance value (i.e., a threshold limit value for occupational exposure), the benchmark dose (BMD) method was used [9,10]. In this phase, the probabilistic approach was applied [4,5,11]. The details of the method used are reported in the supplementary material; below only some key points are presented. The Benchmark Dose Software v. 3.2 (“BMDS” U.S. Environmental Protection Agency, Washington, DC, USA) [12] was used to estimate a “benchmark concentration” (BMC) and the respective lower (BMCL) and upper bounds (BMCU). The benchmark response for continuous data was defined as the change in the mean equal to one standard deviation (SD) in the control group. All the reliable model outputs were considered and combined by weighting, to define an averaged BMC_a_, respective BMCL_a_ and BMCU_a_ values (i.e., the lower (BMCL) and upper (BMCU) bounds of the BMC confidence interval—where “a” is for “animal”) based on the toxicological animal data defined from the hazard assessment. The lower bound (BMCL_a_) was identified as the starting point to calculate a reference point (RP, to be intended as a health-based guidance value (HBGV) and equivalent to an occupational exposure level (OEL) for the purpose of this study). Thus, the BMCL_a_ was extrapolated to an exposure threshold (BMC_h_, where “h” is for “human”) by applying extrapolation factors [13], namely, interspecies (EF_inter_), intraspecies (EF_intra_) extrapolation factors and a factor representing other sources of uncertainty (UF_i_) from the dose–response assessment Equation (1):

(1) $${\mathrm{BMC}}_{h}=\frac{{\mathrm{BMCL}}_{a}}{{\mathrm{EF}}_{\mathrm{inter}}\times {\mathrm{EF}}_{\mathrm{intra}}\times \mathrm{UF}}$$

To implement the probabilistic functions for each parameter (Monte Carlo simulation, Latin hypercube sampling, 10,000 iterations), a Microsoft Excel add-on software (RiskAMP v.4, Structured Data, LLC, San Francisco, CA, USA) was used.

### 2.3. Exposure Assessment

A recent study [8] conducted a systematic review on occupational exposure assessments to CNTs for the period 2000–2018, with the further aim of assessing the relevance of available quantitative exposure data. Further, the authors evaluated and graded the quality of the reviewed studies. Starting from this review study, recent studies from peer-reviewed literature regarding occupational exposure assessments for CNTs were selected for the present study. Only studies classified as of high-quality and with suitable exposure data for occupational exposure to CNTs were considered. More in detail, other than the quality classification performed by Guseva Canu et al. [8], the relevant studies were selected using these further criteria. Only studies published from 2015 to present were included. A proper exposure time period and complete statistics of the measurement data were required, but no restrictions on the measurement metrics were applied. Based on the obtained exposure data, a probability distribution function was assumed for each exposure scenario (ES) or each activity described in the identified ES by means of Monte Carlo simulations (using the same methods described in the previous section).

### 2.4. Risk Characterization and Uncertainty Analysis

The ratio of the exposure (EXP_i_) and the exposure threshold (BMC_h_) distributions (i.e., the risk characterization ratio—RCR), calculated as defined by Equation (2), by sampling the EXP and BMC_h_ distributions over 10,000 Monte Carlo simulations, was used to calculate the likelihood of occurrence of a relevant occupational risk. Intuitively, a risk probability is observed when the exposure value exceeds the BMC_h_ value (i.e., when RCR ≥ 1)

(2) $$\mathrm{RCR}=\frac{{\mathrm{EXP}}_{i}}{{\mathrm{BMC}}_{h}}$$

Based on a previous study [5], the nominal range sensitivity analysis was selected to estimate the level of uncertainty in the RA process, which may have been introduced by the use of models, surrogate data and other assumptions (i.e., lack of relevant data for measured exposure and toxicological profiles). Results are expressed as average contributions (percentage) to the uncertainty in the RCR. Further details were reported in the Supplementary Material.

## 3. Results

### 3.1. Hazard Identification

A recent review study [14] discussed the toxicity of CNTs. Although many studies have been conducted on exposure to CNTs, there are still many uncertainties and gaps related to the identification of the hazard in all its phases, and further research is required [8,14]. According to the data collected from the literature on the experimental analysis of animals and pulmonary toxicity, the possible effects related to exposure from inhaled NTC are different: persistent inflammation, pulmonary fibrosis and lung cancer [14]. Many studies record persistent inflammation of the respiratory system with a tendency to inflammation caused already at low concentrations of inhaled material. According to some studies, exposure to MWCNT (of different types) by inhalation shows a persistence of inflammation with a NOAEL between 0.1 and 0.25 mg/m^3^ and a LOAEL equal to 0.2 mg/m^3^ [15,16,17,18]. To date, many studies have shown pulmonary fibrosis associated with persistent lung inflammation of the pulmonary organ, but there is no clear evidence of a direct relationship between the disease and exposure to CNTs. There is no evidence that can confirm persistent hyperplasia induced by exposure to CNTs. It would also seem that short-length fibers are less persistent than long ones and that the type and size can influence the body’s response to exposure to these materials. There is evidence for which CNTs have high biopersistence, but that shorter fibers have a lower half-life. Studies conducted on inhalation exposure to MWCNTs with a geometric mean length of 1.1 µm reveal a biological half-life with 0.37 mg/m^3^ of 51–55 days [19]. The insolubility of these materials is comparable, if not greater, to that of asbestos. However, some CNTs become more soluble if, following degradation, they reduce their length and possibly their biopersistence, thus allowing them to be removed from the lungs [20]. Many studies show genotoxicity following acute exposure to CNTs which can induce, consequently, damage to DNA, micronuclei and mutations of lung cells; the damage to DNA strands is dose-dependent on the inhaled dose of CNTs [14]. There is minimal evidence relating inhalation exposure to CNT to tumor pathology. The study by Kasai and collaborators [21] demonstrates the presence of malignant tumors affecting the respiratory system. The long-term inhalation study (104 weeks) highlights the onset of cancer of the respiratory system with an increase in the pathology at concentrations of inhalation exposure starting from 0.2 and 2 mg/m^3^ for males and females, respectively. The NOAEL value was found to be 0.02 mg/m^3^. A further study estimated the carcinogenic effects of CNTs: the study was conducted for 15 days, and results highlighted an increase in the tumor rate (bronchial alveolar adenoma and adenocarcinoma) and suggests CNTs as promoters of carcinogenesis [22]. However, although researchers have compared some forms of CNTs to other hazardous fibrous materials, such as asbestos [20,23], the possible health effects associated with CNT exposure are still to be defined. A particular MWCNT (Mitsui-7) was classified as “possibly carcinogenic to humans” (Group 2B), while for other CNTs, insufficient data are available for classification [8,24]. Regarding developmental and reproductive toxicity, limited data are available, and their interpretation is difficult [25]. In summary, (i) inhalation exposure to CNTs could be associated with several adverse effects both in animals and in humans (adverse effects identified in humans are similar to the toxicological endpoints identified in in vivo animal studies); (ii) to date, there is no evidence of causal inference between these effects and CNT exposures [3]. In conclusion, from the first phase of the RA process related to inhalation exposure to CNTs there are criticalities of investigation and the definition of the hazard itself. The available studies do not consider all the variables of the material, such as the different sizes and types of the fibers that compose them. Overall, the lack of an appropriate and standardized method of investigation does not facilitate the hazard assessment [8,14].

### 3.2. Dose–Response Assessment

A health-based guidance value for occupational inhalation exposure to CNTs was obtained by means of the benchmark dose method. Starting from published inhalation exposure tests [14] (Table S1, Supplementary Material), some of the reference studies were selected. Although affected by a certain degree of uncertainty, this information was used in the following phases of the risk assessment. A total of 25 reliable models were fitted to the dose–response data based on mass concentration metrics (Table S2, Supplementary Material). No reliable mathematical model based on number concentration metrics was obtained. Then, models averaging an overall BMCL_a_ log-normal distribution (*p* < 0.01; Kolmogorov–Smirnov test) were obtained, with a mean of 9.451 ± 1.006 mg/m^3^ to allow a stochastic BMC_h_ calculation (Table 1). After applying uncertainty factors over 10,000 Monte Carlo simulations, a BMC_h_ (i) was described as a lognormal distribution with a GM of 10 µg/m^3^ and a GSD of 1.5 µg/m^3^. This BMC_h_ value was considered equivalent to an OEL value for the subsequent phase of risk characterization in the framework of the present study.

In terms of comparison, an 8 h time weighted average (TWA) recommended exposure limit (REL) for CNTs and carbon nanofibers of 1 µg/m^3^ (of Elemental Carbon as respirable mass concentration) was proposed by the National Institute for Occupational Safety and Health (NIOSH) [26]. An exposure limit of 0.01 fibers/cm^3^ was proposed by the British Standards Institute [27]. Finally, a no-observed adverse effect level (NOAEL) of 0.98 mg/m^3^ (from a 13 week inhalation study in rats) was obtained in a previous study. From this, an Occupational Exposure Level (OEL) of 6 µg/m^3^ was estimated for MWCNT [28]. It should be noted that the value proposed in this study is described as a distribution of values, which, therefore, includes a rather large scale of values that is comparable overall with the above-mentioned reference values.

### 3.3. Exposure Assessment

Only one suitable study was found for the present analysis [29], after selection of data. In the selected study, workers’ exposure to SWCNTs was assessed during the production of conductive films. Three different work events (WEs) were assessed: (WE1) manufacturing of SWCNT films during normal operation using local exhaust ventilation 9 LEV); (WE2) manufacturing of SWCNT films without LEV; (WE3) cleaning of one of the reactors, by means of different measurement, namely, “MT1”: mobility particle size distributions were measured in 13 channels from 10 to 420 nm with an electrical mobility spectrometer; “MT2”: aerodynamic particle size distributions were measured from 7 nm to 10 μm in 13 stages with an electrical low-pressure impactor; and “MT3”: optical particle size distributions were measured in 16 channels from 0.3 to 10 μm with an Optical Particle Sizer. Exposure data were also presented for working hours (WH) (i.e., during weekdays mainly between 08:00 and 17:00) and for the non-working hours (NWH). Further details on the selected exposure scenario and on measurement techniques are available in the Supplementary Material. Exposure data to CNTs, expressed as mass concentrations [µg/m^3^] for the three WE are summarized in Table 2 (mean and standard deviation (SD)). Monte Carlo simulations were used to simulate a probability lognormal distribution function for exposure in each WE (Kolmogorov–Smirnov test; *p* < 0.01). Table 3 presents the summary of results of the simulations.

The geometric means for exposure ranged from 0.53 (MT2, WE1) to 24.8 µg/m^3^ (MT2, WE2), with lower average values for the NWH periods, which can be considered as representative of background values. It is necessary to observe that the concentrations reported in Table 2 and Table 3 should be considered with caution. In fact, the direct-reading instruments adopted in the reference study may have detected SWCNTs in the form of larger particles (>300 nm) even if unable to directly identify SWCNT emissions. The sampling and characterization of SWCNTs by Transmission Electron Microscopy (TEM) was also performed, which allowed us to confirm the presence of airborne SWCNTs. Therefore, calculated particle mass (assuming SWCNTs bunch with density = 1 g/cm^3^, diameter = 20 nm; length = 10 μm; aspect ratio ~500) was also presented and discussed in the study. Thus, although the TEM analysis technique is considered to be more accurate and may provide results with a metric (i.e., number concentration of CNT) deemed better for the purposes of risk assessment, for the purposes of this discussion, the data obtained by means of mass concentration estimation were considered. However, the estimated CNT mass concentrations were adopted to represent the exposure in this risk assessment study, although fiber counts would be more specific. This choice was made both because it was not possible to derive a BMC_h_ value for the CNTs and to ensure a precautionary approach (therefore, overestimating the exposure to CNTs). With the same purpose, the estimated exposure values for WE1-3 will be considered, without subtracting the background value (NWH).

### 3.4. Risk Characterization and Uncertainty Analysis

Results of the risk characterization are reported in Table 4 for the three exposure scenarios. WE1, WE2 and WE3 were characterized by means of different MTs. WE characterized by means of MT1 resulted in distributions with RCR < 1. WEs assessed by means of MT3 resulted generally in a low level of risk (very close to 0%), and none of the WEs showed a statistically significant level of risk at the 95% CI. On the contrary, WEs investigated by means of MT2 resulted in a higher level of risk (always with a probability > 5% of obtaining RCR ≥ 1), though only WE2 resulted in a significant RCR ≥ 1 at a 95% CI (98.6% of the estimated RCR indicated a possible risk to the exposed workers).

Overall, WE1 resulted in RCR distributions ≥ 1 (i.e., risk present) with a likelihood between 0 and 7.0%. None of the three exposure profiles had a statistically significant level of risk measured at a 95%CI. Therefore, ~93 to 99.9% of the estimated exposures were at RCR < 1. Similarly, WE3 and WH resulted in RCR distributions ≥ 1 with a percentage between 0 and about 16.2%. Additionally, in this case, none of the three exposure profiles had a statistically significant level of risk measured at a 95% CI. Therefore, ~84–99.9% of the estimated exposures were at RCR < 1. WE2 resulted in RCR distributions ≥ 1 with a percentage between 0 and 98.6%. One of three WE had a statistically significant level of risk at a 95% CI. Higher exposure estimates and, consequently, RCR values were obtained when WEs were investigated by means of MT2. Interestingly, in a source study for this scenario [29], the authors outlined that the potential release of SWCNTs during collection chamber openings could be expected, both while using LEV (WE1) and without using LEV (WE2). Further, the release of the SWCNTs may occur while performing cleaning operations with wet wipes (WE3). During WE1 and WE3, exposure levels were well below the proposed OEL (1.0 × 10^−2^ fibers/cm^−3^), and during the WE2, it was clearly exceeded (5.6 SWCNTs/cm^3^). Further, in terms of calculated particle mass, the exposure associated with SWCNT manufacturing with LEV (WE1) would not have exceed the recommended OEL of 1 μg/m^3^. Contrarywise, exposure measured for manufacturing without LEV (WE2) was equal to 20.5 μg/m^3^ (assuming the average mass concentration of NWH (NWH = 4.5 μg/m^3^) as the background and subtracting it from the average mass concentration of WE2, (25.0 μg/m^3^)), which exceeds the NIOSH OEL. Similarly, exposure associated with the reactor cleaning (WE3 = 6.1 μg/m^3^) resulted in a higher value than the proposed NIOSH OEL. Overall, the obtained results suggest that the workplaces may be influenced by the presence of other airborne nanomaterials or nanoparticles than CNTs and that the estimation of CNT mass concentration represented a precautionary approach. It is also worth noting that the measured exposure values (and consequently the calculated RCR values) show a certain, and relevant, variability depending on the measurement technique used for monitoring exposure. Therefore, the importance of an exposure assessment conducted with proper methods and rigorous criteria for risk assessment purposes is recalled. It should also be noted that risk management and mitigation measures were already defined in the investigated working environment [29]. Despite this, the proposed probabilistic approach outlined the potential presence of a level of risk (although not always significant) and allowed us to estimate the probability that this risk will occur in the considered WEs, even in conditions for which this had not been previously identified with the classical deterministic approach. The level of risk could be reduced by further implementing risk management measures and protocols. A typical hierarchical risk management approach based on engineering controls, procedures and personal protective equipment could be implemented. Specific work tasks (i.e., WE2) are associated with non-negligible occupational exposure; however, the task described in WE2 did not consider the use of LEV. This confirms the importance of the development of appropriate procedures for the workplace, and that worker monitoring and the correct use of LEV, other than education and training of workers, could be implemented to prevent workplace contamination and reduce workers’ exposure.

### 3.5. Limitations of the Sudy

The major limitations of the present study (which should be considered in the interpretation of the results and the general applicability of the findings) are summarized hereafter: (i) First, CNTs have a wide range of properties, which also affect CNTs’ toxicological effects. CNTs constitute a very heterogeneous family, and vary a lot even within subfamilies, such as single wall CNT (SWCNT) or multiwall CNTs (MWCNT). These features pose an immediate problem for the risk assessment of this material due to variability in physicochemical properties, toxicological effects or release/exposure potentials. For the present study, no particular constraints were placed on the selection of toxicological studies, regarding the characterization of CNTs, trying to best represent this potential variability. Moreover, (ii) the use of parametric distribution simulations implies that certain assumptions and simplifications introduced uncertainties into the RA process. Hence, to quantify the impact of this uncertainty on the overall results, a nominal range sensitivity analysis (SA) was performed. The SA was performed considering each possible determinant: results, expressed as an average percent contribution to the uncertainty in the RCR estimates, are consistent with previous studies [4] and outlined that the major part (>75%) of the variation in the RCR distributions was influenced by uncertainties in the factors applied to the BMCh calculation (i.e., EFinter (25.2%) EFintra (29.1), and UFi (23.1)), rather than in the exposure (22.1%) and BMCLa (0.5%) estimations. A further bias in the risk assessment process could have been introduced with (iii) the selection of sources used for the hazard assessment. Nevertheless, available studies were carefully considered with a precautionary approach. Furthermore, (iv) the criteria used in the dose–response assessment phase could have introduced a certain order of bias, as reported in a previous study [5]. Nevertheless, the adopted precautionary and probabilistic approach is expected to contribute to reducing the effect of this limitation. Having used data obtained from tests on MWCNTs in the hazard identification phase contributes to conferring a precautionary aspect to the study, as an effect at lower doses is expected for this type of CNT [8,14]. Further, (v) it should be noted that risk assessment of engineered nanomaterials should use a dose-metric that is the best indication of toxicity as well as exposure. However, no valid mathematical model fitted to the dose–response data based on number concentration. This clearly prevented the proposed approach from being applied to exposure values expressed with this metric, limiting its applicability to scenarios where exposure was measured or estimated as a mass concentration. Further, the adopted approach was based on mass concentration as metric; this is contrasting with the benchmark exposure limit adopted in some countries, based of CNT-fiber count, and sustains the question around the toxicological relevance of different CNT exposure metrics [8]. In this regard, (vi) differences could be observed by comparing exposure levels expressed as mass or fiber-count metric with the respective thresholds. This could result, for the same WE, in defining different probabilities of risk. Thus, performing a multimetric risk assessment is always recommended. Finally, (vii) exposure data were taken from a high-quality published study: nevertheless, scenario-specific variables may not have been considered (e.g., use of personal protective equipment and measurement locations) for the purposes of the applicability of the probabilistic model. Further, some other valuable studies could have been excluded from this study due to the strict inclusion/exclusion criteria.

## 4. Conclusions

The probabilistic approach was applied for a risk assessment study, concerning occupational exposure to CNTs under different exposure conditions. This allowed us to estimate a specific threshold limit value for occupational exposure to CNTs and to quantitatively define the probability of a risk for each investigated exposure situation. Despite some limitations, this study allowed us: (i) to obtain a benchmark concentration (expressed as mass concentrations) for occupational exposure to CNTs; (ii) to include the uncertainty in the source data; and (iii) to quantitatively estimate the likelihood of risk for a selected occupational scenario. The evidence obtained in this study can also be applied to further case studies and exposure scenarios, although, as mentioned, a certain level of caution must be used in extending the results of this study to other scenarios or to particular types of CNTs. In practice, it would be possible to use this same approach, but based on a stricter, more focused number of dose–response studies (of adequate quality), to better adapt the assessment and provide a better match with the properties of a particular type of CNT. However, the results obtained in this study will have to be updated in light of new information that will become available, especially as regards hazard identification and exposure assessment of CNTs. One of the major issues concerns the uncertainty related to chronic health effects, including whether some types of CNTs may be carcinogenic (possibly with an asbestos-like mechanism). Thus, continued efforts are needed to reduce exposures as much as possible, while robust and cautelative occupational exposure limits must be defined.

## Figures and Tables

**Table 1:** BMC_a_, BMCL_a_, BMCU_a_ (GM ± GSD; mg/m^3^) and BMCh (GM ± GSD) calculated for carbon nanotubes.

BMC_a_	BMCL_a_	BMCU_a_	BMC_h_
GM (± GSD) [mg/m^3^]			GM (± GSD) [µg/m^3^]
14.1 (± 1.1)	9.4 (± 1.0)	20.4 (± 2.3)	10.0 (± 1.5)

**Table 2:** Exposure to carbon nanotubes expressed as mass concentrations [µg/m^3^] for the three selected work events (WEs), characterized by means of different measurement techniques (MTs). The data are presented as mean and standard deviation (GSD). Note: n.a. = not available.

MTs	WEs	Exposure [µg/m^3^]	
		Mean	SD
MT1	WE1	0.83	0.18
	WE2	1.0	0.07
	WE3	1.2	0.12
	WH	1.2	0.32
	NWH	0.92	0.31
MT2	WE1	3.4	22
	WE2	25	3.80
	WE3	6.1	12.0
	WH	6.7	22.0
	NWH	4.5	6
MT3	WE1	0.81	0.82
	WE2	1.7	0.69
	WE3	n.a.	n.a.
	WH	1.5	5.7
	NWH	0.42	0.21

**Table 3:** Descriptive statistics for results of the 10,000 Monte Carlo simulations used to estimate the distribution of exposure values for the considered work events (WEs), characterized by means of different measurement techniques (MTs). Exposure to CNTs was expressed as mass concentrations [µg/m^3^]. The data are presented as geometric mean (GM), geometric standard deviation (GSD), minimum and maximum values (min, max) and 5th and 95th percentiles. Note: n.a. = not available.

MTs	WEs	Exposure [µg/m^3^]					
		GM	GSD	Min	5th Percentile	95th Percentile	Max
MT1	WE1	0.81	1.24	0.38	0.57	0.62	1.71
	WE2	1.00	1.07	0.77	0.89	0.91	1.29
	WE3	1.19	1.10	0.83	1.01	1.05	1.71
	WH	1.16	1.30	0.46	0.76	0.82	3.23
	NWH	0.87	1.39	0.23	0.51	0.57	3.22
MT2	WE1	0.53	7.08	0.00	0.02	0.04	1287
	WE2	24.8	1.16	14.49	19.44	20.4	44.5
	WE3	2.76	3.53	0.01	0.35	0.53	372
	WH	2.65	2.76	0.002	0.15	0.26	493
	NWH	2.65	2.76	0.06	0.49	0.71	89.8
MT3	WE1	0.56	2.30	0.02	0.15	0.19	12.8
	WE2	1.58	1.48	0.33	0.83	0.95	6.81
	WE3	n.a.	n.a.	n.a.	n.a.	n.a.	n.a.
	WH	0.38	5.10	0.001	0.03	0.04	137
	NWH	0.38	1.60	0.05	0.17	0.20	2.39

**Table 4:** RCR distributions for WE1-3 and WH (10,000 Monte Carlo simulations). RCR distributions were calculated considering mass concentrations [µg/m^3^] as metrics for exposure and BMC_h_ distributions. The data are presented as geometric mean (GM), geometric standard deviation (GSD), minimum and maximum values (min, max) and lower and upper limits of the confidence interval of the mean (95% CI). Note: n.a. = not available.

MT	WE	Risk Characterization Ratio							
		GM	GSD	Min	5th Percentile	95th Percentile	Max	RCR ≥ 1 (95% CI)	Probability (%) RCR ≥ 1
MT1	WE1	0.08	1.54	0.02	0.08	0.08	0.35	no	0.0
	WE2	0.10	1.47	0.03	0.10	0.10	0.42	no	0.0
	WE3	0.12	1.48	0.03	0.12	0.12	0.48	no	0.0
	WH	0.12	1.58	0.02	0.11	0.12	0.98	no	0.0
MT2	WE1	0.05	7.37	0.00003	0.05	0.05	147	no	7.0
	WE2	2.47	1.50	0.53	2.45	2.49	10.7	yes	98.6
	WE3	0.28	3.72	0.001	0.27	0.28	73.7	no	16.2
	WH	0.20	5.05	0.0002	0.20	0.21	72.8	no	16.4
MT3	WE1	0.56	2.49	0.002	0.55	0.57	2.0	no	0.1
	WE2	0.16	1.72	0.03	0.16	0.16	1.42	no	0.1
	WE3	n.a.	n.a.	n.a.	n.a.	n.a.	n.a.	n.a.	0.0
	WH	0.04	5.31	0.0001	0.04	0.04	21.0	no	2.4

## Data Availability

The data presented in this study are available on request from the corresponding author.

## Supplementary Materials

The following Supplementary Materials are available online at https://www.mdpi.com/2079-4991/11/2/409/s1: Further details about methods and results; Table S1. In vivo toxicity of Carbon Nanotubes (CNTs); Table S2. BMC, BMCL, BMCU and AIC results for a change in the mean equal to one control SD for the selected parameter. Dosimetry: mass concentrations (mg/m^3^). Only results of viable models are shown.

## Abbreviations

95% CI	95% confidence interval
AIC	Akaike’s information criterion
AM	arithmetic mean
BMC	benchmark concentration
BMC_h_	benchmark concentration-human
BMCL	benchmark concentration (95% CI Lower Bound)
BMCU	benchmark concentration (95% CI Upper Bound)
BMD	benchmark dose
BMDL	benchmark dose (95% CI lower bound)
BMDU	benchmark dose (95% CI upper bound)
CNTs	Carbon nanotubes
EF_inter_	interspecies extrapolation factor
EF_intra_	intraspecies extrapolation factor
ES	exposure scenario
EXP	exposure data distribution
GM	geometric mean
GNPs	graphene nanoplatelets
GSD	geometric standard deviation
HBGV	health-based guidance value
LOAEL	lowest-observed adverse effect level
MN	manufactured (engineered) nanomaterial
MWCNTs	multiwalled carbon nanotubes
MT	measurement technique
NOAEL	no-observed-adverse-effect level
NWH	non-working hours
OEL	occupational exposure level
RA	risk assessment
RCR	risk characterization ratios
REL	recommended exposure limit
SD	standard deviation
SWCNTs	single-walled carbon nanotubes
TWA	time-weighted average
UF_i_	uncertainty factor
WE	work events
WH	working hours
