# Supplementary material for: Carbon Nanotubes: Probabilistic Approach for Occupational Risk Assessment
Source: Nanomaterials (Basel). 2021 Feb 5;11(2):409. doi: 10.3390/nano11020409 (PMC7916016; doi:10.3390/nano11020409)
Supplement: Supplementary file 1 [file nanomaterials-11-00409-s001.pdf]

nanomaterials

Supplementary Materials

Article

Carbon Nanotubes: Probabilistic Approach for Occupational Risk Assessment

Andrea Spinazzè *, Carolina Zellino, Francesca Borghi, Davide Campagnolo, Sabrina Rovelli, Marta Keller, Giacomo Fanti, Andrea Cattaneo and Domenico M. Cavallo

Dipartimento di Scienza e Alta Tecnologia, Università degli Studi dell'Insubria, Via Valleggio, 11–22100 Como, Italy; carolinazellino@gmail.com (C.Z.), f.borghi2@uninsubria.it (F.B.); davide.campagnolo@uninsubria.it (D.C.); sabrina.rovelli@uninsubria.it (S.R.); mkeller@uninsubria.it (M.K.); giacomo.fanti@uninsubria.it (G.F.); andrea.cattaneo@uninsubria.it (A.C.); domenico.cavallo@uninsubria.it (D.M.C.)
* Correspondence: andrea.spinazze@uninsubria.it

Supplementary Material

Table S1. In vivo toxicity of carbon nanotubes (CNTs)

Exposure	CNT type	CNT characterization	Animal model	Dose, route and duration of exposure	Post exposure period	Results	Reference
Inhalation	MWCNT mixture, MWCNT and graphene nanofibers	Dimension: 10–20 nm×5-15 μm<br>Impurity: 0.5% Ni and Fe<br>Surface Area: 100 m^2/g<br>MMAD: 700–1000 nm/1800 nm	Male Mouse C57BL/6	0.0; 0.3; 1.5mg/m^3<br>7 e14 days<br>6 h/day	Day 0	No inflammation, damaged tissue, significant pathologies.<br>No changes in gene expression in the lungs.<br>Non-monotonic systemic immune suppression.<br>Estimated dose deposited 0.2, 0.5, 2.7 mg/kg per 0.3, 1, 5 mg/m^3.	[1]
Inhalation	MWCNT mixture, MWCNT and graphene nanofibers	Dimension: 10–20 nm×5-15 μm<br>Impurity: 0.5% Ni and Fe<br>Surface Area: 100 m^2/g<br>MMAD: 700–1000 nm/1800 nm	Mouse C57BL/6 Male	0.0; 0.3; 1 mg/m^3<br>14 days<br>6 h/day	Day 0	Systematic immunosuppression given by the release of signals from the lung and not by the systemic uptake of CNT.	Mitchell et al. (2009)
Inhalation	MWCNTs	Dimension: 5–15nm×0.1-10 μm<br>Impurity: 10% metal oxide<br>Surface Area: 250–300 m^2/g<br>MMAD: 0.5–1.3 μm	Wistar rat	0; 2; 8; 32 mg/m^3<br>5 consecutive days<br>6 h/day<br><br>0.0; 0.1; 0.5; 2.5 mg/m^3.<br>13 weeks 6 h/day per 5 consecutive days	8, 28 days<br><br>3, 24 days	Multifocal granulomatous inflammation, severe diffuse pulmonary histiocytosis with influx of neutrophils and bronchoalveolar hyperplasia at 8 and 32 mg/m^3. NOEC not established<br><br>No systemic toxicity.<br>Lung weight gain pronounced multifocal granulomatous inflammation, histiocytic	[2]

						inflammation and diffuse neutrophilia. Intra-alveolar lipoproteinosis in the lungs and associated lymph nodes.	

Table S1 (continue)

Exposure	CNT type	CNT characterization	Animal model	Dose, route and duration of exposure	Post exposure period	Results	Reference
Inhalation	MWCNTs	Co: 0.46–0.53%<br>BET: 253 m^2/g<br>Length: 200–300 nm	Wistar rat Male and Female	0.1; 0.4; 1.5; 6 mg/m^3.<br>13 weeks<br>6 h/day per 5 consecutive days	6 months	- The pathological changes induced are consistent with the phenomena related to overload.<br>- The etiopathological sequence of the inflammatory events caused appears to be related to the high volume of displacement of the low-density CNT assembly structure rather than to any still poorly defined intrinsic toxic property.<br>- Inflammation at 0.4 mg/m^3 (transient); 1.5 mg/m^3 (persistent); 6 mg/m^3 (persistent)	[3]
Inhalation	Carbon nanofibers	Carbon: >99.5%<br>Diameter: 158 nm<br>Length: 5.8 μm<br>BET: 13.8 m^2/g	Sprague Dawley rat Male and Female	0.0; 0.54 mg/m^3 (4.9 f/cc)<br>2.5 mg/m^3 (56 f/cc)<br>25 mg/m^3 (252 f/cc)<br>13 weeks<br>6 h/day per 5 days	90 days	- NOAEL 0.54 mg/m^3, 4.9 f/cc).<br>- Persistent inflammation at 25 mg/m^3.	[4]

Inhalation	MWCNTs	Diameter: 44 nm<br>BET: 69 m²/g<br>Fe: 0.0005%	Wistar rat Male	0.37 mg/m³ (>70% individuals)<br>4 weeks<br>6 h/day for 5 days	3 days<br>1, 3 months	- Transient inflammation, no granulomatous lesions.	[5]
Inhalation	SWCNTs	Diameter: 3 nm<br>BET: 1064 m²/g<br>Impurity: 0.03%	Wistar rat Male	0.03 mg/m³ (5 * 10⁴ SWNCTs/cc)<br>0.13 mg/m³ (6.6 * 10⁴ SWCNTs/cc)<br>4 weeks; 6 h/day for 5 days	3 days<br>1, 3 months	- No inflammation observed.	[5]

Table S1 (continue)

Exposure	CNT type	CNT characterization	Animal model	Dose, route and duration of exposure	Post exposure period	Results	Reference
Inhalation	MWCNTs	Dimension: 94.1-98nm × 5.53–6.19 µm<br>Purity: >99.6–99.8%<br>Surface Area: 24–28 m²/g<br>MMAD: 1.4–1.6 µm	F344 rat, Male and Female	0.0; 0.2; 1; 5 mg/m³.<br>13 weeks<br>5 days/week<br>6 h/day	Day 0	- LOAEL 0.2mg/m³.<br>- Increased lung weight and inflammatory parameters in BALF.	[6]
Inhalation	MWCNTs	Median dimensional distribution: 376 µm<br>Ash: 8,6%<br>Apparent density di: 0,085 g/cm³<br>Specific Surface: 187 m²/g<br>Metal: (from the catalyst) 3,2 % di Al and 2,7% per Fe	Wistar rats Male and Female	0.05; 0.25 and 1.25 mg/m³<br>6 h/day<br>5 days	24 h<br>28 days	- Macrophages contain phagocytic material with a dose-related increase in incidence; partial recovery at 28 days.<br>- Normal physiological response to the overload of insoluble and non-adverse particles.	[7]
				0.05; 0.25 and 5.0 mg/m³ 5 days/week	24 h<br>90 days	- Lung inflammation characteristic of an overload	

	90 days	of insoluble particles at 5.0 mg/m³.<br>- Signs of clearance and recovery at 0.25 mg/m³.<br>- No pulmonary genotoxicity and distally to bone marrow, liver and kidney.<br>- NOAEC 0.25 mg/m³ (0.28 mg/m³ as effective concentration) for repeat dose toxicity.

Notes: MMAD: Mass Median Aerodynamic Diameter; BET: Brunauer Emmett Teller.

Table S2. BMC, BMCL, BMCU, and AIC results for a change in the mean equal to one control SD for the selected parameter. Dosimetry: mass concentrations (mg/m^3). Only results of viable models are shown.

Parameter	Model	BMC [mg/m^3]	BMCL [mg/m^3]	BMCU [mg/m^3]	AIC
Body weight %	frequentist Exponential degree 5 v1.1	0.762	0.261	2.742	-343.0
Absolute weight	frequentist Exponential degree 5 v1.1	0.302	0.259	2.411	33.3
ALKP	frequentist Exponential degree 4 v1.1	3.842	2.370	n.c.	5200.3
Other %	frequentist Exponential degree 3 v1.1	8.307	1.282	n.c.	43.7
BIN %	frequentist Hill v1.1	0.644	0.449	0.895	1042.8
Cell proliferation % (parenchymal)	frequentist Exponential degree 5 v1.1	25.302	2.712	n.c.	-106.4
Cell proliferation % (subpleural)	frequentist Exponential degree 5 v1.1	2.983	2.607	17.716	-217.2
Cell prolif. % (terminal bronchial)	frequentist Polynomial degree 5 v1.1	45.191	26.785	72.948	226.8
GGT	frequentist Hill v1.1	0.191	0.130	0.311	922.3
IL -1b	frequentist Polynomial degree 14 v1.1	7.908	5.055	15.592	1086.3
IL -1b	frequentist Polynomial degree 12 v1.1	7.908	5.062	15.592	1086.3
IL -1b	frequentist Polynomial degree 3 v1.1	7.908	5.240	15.592	1086.3
IL -1b	frequentist Polynomial degree 2 v1.1	7.908	5.297	15.592	1086.3
IL -1b	frequentist Power v1.1	7.908	5.297	15.592	1086.3
IL-1°	frequentist Polynomial degree 7 v1.1	33.795	5.621	n.c.	1237.1

Lactate dehydrogenase	frequentist Polynomial degree 21 v1.1	27.317	25.870	55.793	7070.8
Lung/brain relative weights	frequentist Exponential degree 5 v1.1	12.187	2.589	23.438	2359.2
Macrophages %	frequentist Exponential degree 4 v1.1	1.737	1.350	2.215	4162.7
MTP	frequentist Hill v1.1	2.587	1.887	3.814	1541.3
Neutrophil %	frequentist Hill v1.1	0.925	0.600	1.352	1753.7
PROT	frequentist Exponential degree 4 v1.1	0.302	0.202	0.527	2686.2
Terminal body weights	frequentist Polynomial degree 19 v1.1	26.392	25.543	48.428	4922.4
TNF-a	frequentist Exponential degree 4 v1.1	0.159	0.115	0.239	770.2
Total cells	frequentist Hill v1.1	13.184	6.454	24.885	2506.3
Viability	frequentist Polynomial degree 22 v1.1	78.164	5.361	n.c.	1124.8

Note: ALKP: Alkaline phosphatase; BIN: BIN binucleated macrophages; BMC: benchmark concentration; BMCL: benchmark concentration (95% CI Lower Bound); BMCU: benchmark concentration (95% CI Upper Bound); IL-1a: Interleukin 1 alpha; IL-1b: Interleukin 1 beta; GGT: Gamma-glutamyltransferase; MTP: Microsomal triglyceride transfer protein; PROT: total protein; TNF-a: Tumor necrosis factor; n.c.: not calculated.

2. Methods

2.2. Dose–Response Assessment

The benchmark dose (BMD) method was used to estimate a health-based guidance value (i.e., a threshold limit value for occupational exposure) [8,9]. The term "benchmark concentration" (BMC) was used instead of BMD, to emphasize that the adopted model refers to whole-body concentration data [10]. The estimation of BMCs and the respective lower (BMCL) and upper bounds (BMCU) was performed using the Benchmark Dose Software v. 3.2 ("BMDS" U.S. Environmental Protection Agency, Washington, DC, U.S.A.) [11] and applying rules consistent with BMD modeling guidelines [12]. Only viable model outputs were considered in this study, based on the best-fit model selected according to the decision logic determined prior to modeling. All models specified in the BMD modeling guidelines [12] were used, if appropriate for the specific data type (i.e., continuous dose–response data). The benchmark response was defined as the change in the mean equal to one control standard deviation (SD) for continuous data. The viable models and associated BMCs (with corresponding BMCLs and BMCUs) for each dose–response set were selected according to criteria defined previously (Wignall et al., 2014). Since different dose–response data sets were considered, the lowest BMC and its associated BMCL were selected, regardless of the end-point / effect. It should be noted that the goal was not to find the single best-fitting model, but rather to consider results from all valid models. The individual models' results were combined by weighting (with higher weights for models that showed better fits): the model averaging approach was used to define an AIC-weighted average BMC and respective BMCL and BMCU values, considered as the lower and upper bounds of the BMC confidence interval, respectively. A reference point (RP) also called the health-based guidance value (HBGV) was defined. The average BMC (and corresponding BMCL and BMCU values) calculated by BMDS based on the toxicological animal data defined from the hazard assessment are referred to as BMC_a, $BMCL_a$, and $BMCU_a$ (where "a" stands for "animal"). The lower bound ($BMCL_a$) was used as a starting point to calculate the potential RP as a precautionary approach. The $BMCL_a$ was extrapolated to a human effect threshold referred to as BMC_h

(where “h” stands for "human"), which was considered equivalent to a HBGV or to an occupational exposure level (OEL), by applying extrapolation factors [13], using Equation (1):

$$BMC_h = \frac{BMCL_a}{EF_{inter} \times EF_{intra} \times UF_i} \quad (1)$$

EF_{inter} and EF_{intra} are inter- and intraspecies extrapolation factors, and UF_i contains other sources of uncertainty from the dose–response assessment.

Based on the probabilistic approach defined by previous authors [10,14,15], lognormal distributions for EF_{inter}, EF_{intra}, and UF_i were adopted. The assumption is that these values would be log-normally distributed such that a value of 10 (as typically used in deterministic RA approaches) was one order of magnitude greater than the mean and occurred at the 99th percentile. A Microsoft Excel add-on software package ((RiskAMP v.4, Structured Data, LLC, San Francisco, U.S.A.) was used to supply probabilistic functions for stochastic functionality, along with a Monte Carlo simulation approach with Latin hypercube sampling (10,000 iterations). A probability distribution function was assumed for each parameter.

2.4. Risk Characterization and Uncertainty Analysis

Uncertainty analysis was used to estimate the level of uncertainty in each step of the RA process, where possible sources of uncertainty were the use of (i) surrogate data (e.g., animal toxicology data), (ii) models (e.g., exposure estimates), and (iii) other assumptions (NM-specific sources of uncertainty, due to the lack of relevant data for toxicological profiles, known emissions, and measured exposure). The nominal range sensitivity analysis was selected to quantify the uncertainty; this is a local one-at-a-time (OAT) method where one input variable is modified at a time, while all the others are kept constant. It is important to note that such methods cannot consider interactions between different input parameters; all options of the input parameters were considered equally likely. It is reasonable to assume that uncertainties in the analysis related to omitting interactions were much smaller than the other sources of uncertainties (e.g., using unknown probability distributions of the parameter options) [16]. The nominal range sensitivity results were expressed as average percentage contributions to the uncertainty in the RCR calculation, considering each possible determinant (i.e., distributions of $BMCL_a$, EF_{inter}, EF_{intra}, UF_i, and exposure values).

3. Results and Discussion

3.3. Exposure Assessment

After the selection of data using the inclusion/exclusion criteria, only one study was found to be suitable for the present analysis [17]. In the selected study, workers’ exposure to single-walled carbon nanotubes (SWCNTs) during the production of conductive films in an up-scaling factory was assessed. SWCNTs were produced in a high temperature furnace where CO and iron (Fe) seed particles were introduced. From the reactor where they were synthesized, SWCNTs were directed (i) during collection to the deposition chamber and through a collector filter to the exhaust and (ii) during filter change to the exhaust. When the deposition chamber was open for the change of the collector filter, SWCNTs and by-product gases (mainly CO) were potentially released to the workplace air. Local exhaust ventilation (LEV) was used to prevent emissions. Except during gas-phase SWCNT synthesis, the potential release of SWCNTs during other process stages by re-suspension can be considered to be low. Thus, all SWCNT emissions were assumed to occur during reactor collection chamber opening during normal operation.

Three different work events (WE) were registered and monitored: (WE1) manufacturing of SWCNT films using LEV; (WE2) manufacturing one SWCNT film without LEV; (WE3) cleaning of one of the reactors. The work activity took place during weekdays (mainly between 08:00 and 17:00) which were classified as working hours (WH). Data were also collected for the non-WH including weekend (NWH).

Particulate matter concentrations were monitored by using real-time instruments. In particular, the following measurement techniques (MTs) were adopted:

i. MT1: Mobility particle size distributions were measured in 13 channels from 10 to 420 nm with an electrical mobility spectrometer (NanoScan, SMPS TSI Inc., Model 3910, Shoreview, MN, USA; sample flow rate 0.7 l/min; 105 s scan with 15 s retrace).

ii. MT2: Aerodynamic particle size distributions were measured from 7 nm to 10 μm in 13 stages with an electrical low-pressure impactor (ELPI, Dekati Ltd., Finland, Qs = 9.6 l min/1). Logging time interval was set to 1 s which was averaged to 60 s samples with the ELPIVI 4.0 software for the data analysis using stokes density of 1 g/cm^3.

iii. MT3: Optical particle size distributions were measured in 16 channels from 0.3 to 10 μm with an Optical Particle Sizer (OPS, TSI Inc., Model 3330, Shoreview, MN, USA; sample flow rate 1 l/min; 1 s time resolution).

The geometric means for exposure ranged from 0.53 (MT2, WE1) to 24.8 μg/m^3 (MT2, WE3), with lower average values for the NWH periods, which can be considered as representative of background values. It is necessary to observe that the concentrations reported in Table 1 and Table 2 could not be totally referred to as CNT exposure values. In fact, despite being unable to directly identify SWCNT emissions, the online instruments adopted in the reference study may have detected SWCNTs as larger particles (>300 nm). The collection and analysis of SWCNTs on Transmission Electron Microscopy (TEM) grids was also performed, which was found to be the only direct method to detect SWCNTs in workplace air. However, defining quantitative exposure levels by counting SWCNTs with TEM micrographs was challenging. The TEM analysis was able to confirm the presence of SWCNTs in workplace air. More in detail, there was potential for the release of SWCNTs during collection chamber openings, both while using LEV (WE1) and without using LEV (WE2). There was weak evidence that the release of the SWCNTs may also happen during cleaning operations performed under pressure with wet wipes (WE3). During WE1 and WE3, exposure levels were well below the proposed OEL (1.0×10^{-2} fibers/cm^{-3}), and during the WE2, it was clearly exceeded (5.6 SWCNTs/cm^3). Further, in terms of calculated particle mass (assuming SWCNTs bunch with 20 nm of diameter, 10 μm of length, a density of 1 g/cm^3, and aspect ratio of ~500), SWCNT manufacturing with LEV (WE1) would not have exceeded the recommended OEL of 1 μg/m^3. Contrarywise, for manufacturing without LEV (WE2), assuming the background as the average mass concentration of NWH (NWH = 4.5 μg/m^3) and subtracting it from the average mass concentration of WE2 (25.0 μg/m^3), it suggests that the resulting SWCNT exposure concentration (20.5 μg/m^3) clearly exceeds the proposed OEL. Similarly, concerning the reactor cleaning operation, the average mass concentration of WE3 resulted to be 6.1 μg/m^3, thus higher than the proposed OEL. Overall, this evidence suggests that the workplaces were strongly influenced by the presence of other particles than CNTs and that the estimation of CNT mass concentration represented a precautionary approach. Therefore, although the TEM analysis technique is considered to be more accurate and may provide results with a metric (i.e., number concentration of CNT) deemed better for the purposes of risk assessment, for the purposes of this discussion, the data obtained by means of mass concentration estimation were considered. However, the estimated CNT mass concentrations were adopted as opposed to number concentrations (which would be more specific) to represent the exposure in this risk assessment study. This choice was made both because it was not possible to derive a BMCh value for the CNTs and to ensure a precautionary approach (therefore, overestimating the exposure

to CNTs). With the same purpose, the estimated exposure values for WE1-3 will be considered, without subtracting the background value (NWH).

3.5. Limitations and Strengths

In the dose–response assessment phase, the BMR calculated from a change in the mean equal to one control SD was used, as it is the standard reporting level for each dose–response type and does not necessarily represent equivalent values. However, using a 1 control-group SD change for the continuous end-point results in an overestimated risk of approximately 10% for the proportion of individuals <2nd percentile or >98th percentile of controls for normally distributed effects. It should be noted that the EPA Benchmark Dose Technical Guidance recommends always reporting the estimated BMD associated with the BMR in terms of a difference in means equal to 1 SD. However, one of the weaknesses of this BMR definition is that the associated BMD then depends on study-specific factors. Another limitation of using the 1 SD metric is that the estimate of the associated BMD cannot be translated into an equipotent dose in populations with larger within-group variation. The adopted precautionary (i.e., use of $BMCL_a$ for BMC_h) and probabilistic approach is expected to contribute to reducing the effect of this limitation. Additionally, the fact of having used data obtained from tests on MWCNTs in the hazard identification phase contributes to conferring a precautionary aspect to the study, as an effect at lower doses is expected for this type of CNT [18,19].

References

1. Mitchell, L.A.; Gao, J.; Wal, R. Vander; Gigliotti, A.; Burchiel, S.W.; McDonald, J.D. Pulmonary and systemic immune response to inhaled multiwalled carbon nanotubes. *Toxicol. Sci.* **2007**.
2. Ma-Hock, L.; Treumann, S.; Strauss, V.; Brill, S.; Luizi, F.; Mertler, M.; Wiench, K.; Gamer, A.O.; van Ravenzwaay, B.; Landsiedel, R. Inhalation toxicity of multiwall carbon nanotubes in rats exposed for 3 months. *Toxicol. Sci.* **2009**.
3. Pauluhn, J. Subchronic 13-week inhalation exposure of rats to multiwalled carbon nanotubes: Toxic effects are determined by density of agglomerate structures, not fibrillar structures. *Toxicol. Sci.* **2009**.
4. Delorme, M.P.; Muro, Y.; Arai, T.; Banas, D.A.; Frame, S.R.; Reed, K.L.; Warheit, D.B. Ninety-day inhalation toxicity study with a vapor grown carbon nanofiber in rats. *Toxicol. Sci.* **2012**.
5. Morimoto, Y.; Hirohashi, M.; Kobayashi, N.; Ogami, A.; Horie, M.; Oyabu, T.; Myojo, T.; Hashiba, M.; Mizuguchi, Y.; Kambara, T.; et al. Pulmonary toxicity of well-dispersed single-wall carbon nanotubes after inhalation. *Nanotoxicology* **2012**.
6. Kasai, T.; Umeda, Y.; Ohnishi, M.; Kondo, H.; Takeuchi, T.; Aiso, S.; Nishizawa, T.; Matsumoto, M.; Fukushima, S. Thirteen-week study of toxicity of fiber-like multi-walled carbon nanotubes with whole-body inhalation exposure in rats. *Nanotoxicology* **2015**, *9*, 413–422.
7. Pothmann, D.; Simar, S.; Schuler, D.; Dony, E.; Gaering, S.; Net, J.L.; Okazaki, Y.; Chabagno, J.M.; Bessibes, C.; Beausoleil, J.; et al. Lung inflammation and lack of genotoxicity in the comet and micronucleus assays of

industrial multiwalled carbon nanotubes Graphistrength© C100 after a 90-day nose-only inhalation exposure of rats. *Part. Fibre Toxicol.* **2015**, *12*, 1–28.

8. Davis, M.E.; Hart, J.E.; Laden, F.; Garshick, E.; Smith, T.J. A retrospective assessment of occupational exposure to elemental carbon in the U.S. Trucking Industry. *Environ. Health Perspect.* **2011**, *119*, 997–1002.
9. Hardy, A.; Benford, D.; Halldorsson, T.; Jeger, M.J.; Knutsen, K.H.; More, S.; Mortensen, A.; Naegeli, H.; Noteborn, H.; Ockleford, C.; et al. Update: use of the benchmark dose approach in risk assessment. *EFSA J.* **2017**, *15*.
10. Tsang, M.P.; Hristozov, D.; Zabeo, A.; Koivisto, A.J.; Jensen, A.C.Ø.; Jensen, K.A.; Pang, C.; Marcomini, A.; Sonnemann, G. Probabilistic risk assessment of emerging materials: case study of titanium dioxide nanoparticles. *Nanotoxicology* **2017**, *11*, 558–568.
11. U.S. EPA Benchmark Dose Tools | US EPA Available online: https://www.epa.gov/bmds (accessed on Sep 1, 2020).
12. EPA Benchmark Dose Technical Guidance | Risk Assessment | US EPA Available online: https://www.epa.gov/risk/benchmark-dose-technical-guidance (accessed on Sep 1, 2020).
13. ECHA *Guidance on information requirements and chemical safety assessment Chapter R.8: Characterisation of dose [concentration]-response for human health*; 2012;
14. Spinazzè, A.; Cattaneo, A.; Borghi, F.; Del Buono, L.; Campagnolo, D.; Rovelli, S.; Cavallo, D.M. Probabilistic approach for the risk assessment of nanomaterials: A case study for graphene nanoplatelets. *Int. J. Hyg. Environ. Health* **2019**, *222*, 76–83.
15. Slob, W.; Bakker, M.I.; Biesebeek, J.D. ir. Te; Bokkers, B.G.H. Exploring the uncertainties in cancer risk assessment using the integrated probabilistic risk assessment (IPRA) approach. *Risk Anal.* **2014**.
16. Riedmann, R.A.; Gasic, B.; Vernez, D. Sensitivity analysis, dominant factors, and robustness of the ECETOC TRA v3, Stoffenmanager 4.5, and ART 1.5 occupational exposure models. *Risk Anal.* **2015**, *35*, 211–225.
17. Fonseca, A.S.; Viitanen, A.K.; Koivisto, A.J.; Kangas, A.; Huhtiniemi, M.; Hussein, T.; Vanhala, E.; Viana, M.; Querol, X.; Hameri, K. Characterization of exposure to carbon nanotubes in an industrial setting. *Ann. Occup. Hyg.* **2015**, *59*, 586–599.
18. Kobayashi, N.; Izumi, H.; Morimoto, Y. Review of toxicity studies of carbon nanotubes. *J. Occup. Health* **2017**, *59*, 394–407.
19. Guseva Canu, I.; Batsungnoen, K.; Maynard, A.; Hopf, N.B. State of knowledge on the occupational exposure to carbon nanotube. *Int. J. Hyg. Environ. Health* **2020**, *225*, 113472.
